# Lymphoglandular Complex-Like Colorectal Carcinoma Arising in Tubular Adenoma: A Case Report

**DOI:** 10.7759/cureus.103647

**Published:** 2026-02-15

**Authors:** Ping Shi, Xiaobang Hu

**Affiliations:** 1 Department of Pathology and Laboratory Medicine, Penn State College of Medicine, Penn State Health Milton S. Hershey Medical Center, Hershey, USA

**Keywords:** adenoma involving lymphoglandular complexes, histologic features, lymphoglandular complex-like carcinoma, misplacement of adenomatous epithelium, pseudoinvasion

## Abstract

Lymphoglandular complex-like colorectal carcinoma (LGCC) is characterized by invasive tumors confined entirely by lymphoid stroma and is very rare. Histologically, it can closely mimic adenomatous polyps involving lymphoglandular complexes (LGCs) or pseudoinvasion/misplacement of adenomatous epithelium. Here, we report a 57-year-old patient who underwent routine surveillance colonoscopy and was found to have a 30 mm sessile polyp in the ascending colon. The polyp was removed by endoscopic mucosal resection. Initial histologic sections show fragments of tubular adenoma. Due to the large size of the polyp, additional levels were ordered. At the second level of the largest fragment, a small focus of lymphoid aggregate is seen beneath the muscularis mucosa, and there is a small focus of tumor glands (< 1 mm) within this lymphoid aggregate. These glands are completely surrounded by the lymphoid tissue and show an infiltrative pattern. There is no interposed lamina propria. The glands exhibit haphazard distribution, gland angulation with luminal debris, and scattered single cells. The tumor cells show high-grade cytologic atypia. CDX2 immunostain highlighted the tumor glands and single cells, supporting the diagnosis of LGCC. Our case shows that LGCC can be diagnostically challenging. Awareness of its morphologic features can help to avoid diagnostic pitfalls.

## Introduction

Lymphoid aggregates are scattered throughout the small intestine and colon. They can locate above the muscularis mucosae in the lamina propria or immediately beneath the muscularis mucosae within the upper submucosa. These lymphoid aggregates frequently maintain close association with the overlying surface epithelium, a relationship that forms the basis of the term lymphoglandular complexes (LGCs) [1]. Although uncommon, colorectal adenomatous polyps may show dysplastic epithelial involvement of LGCs, a phenomenon that can closely mimic invasive adenocarcinoma when these complexes are situated within the submucosa and represent a distinct form of pseudoinvasion [1].

Lymphoglandular complex-like colorectal carcinoma (LGCC) is a very rare variant of colorectal adenocarcinoma. It is characterized by malignant glands entirely enveloped by prominent lymphoid aggregate, creating a striking resemblance to benign LGCs [2]. Here we described a case of ascending colon polyp harboring LGCC and examined its histomorphologic features to aid in recognition of this diagnostic challenging entity.

## Case presentation

**Figure 1:**
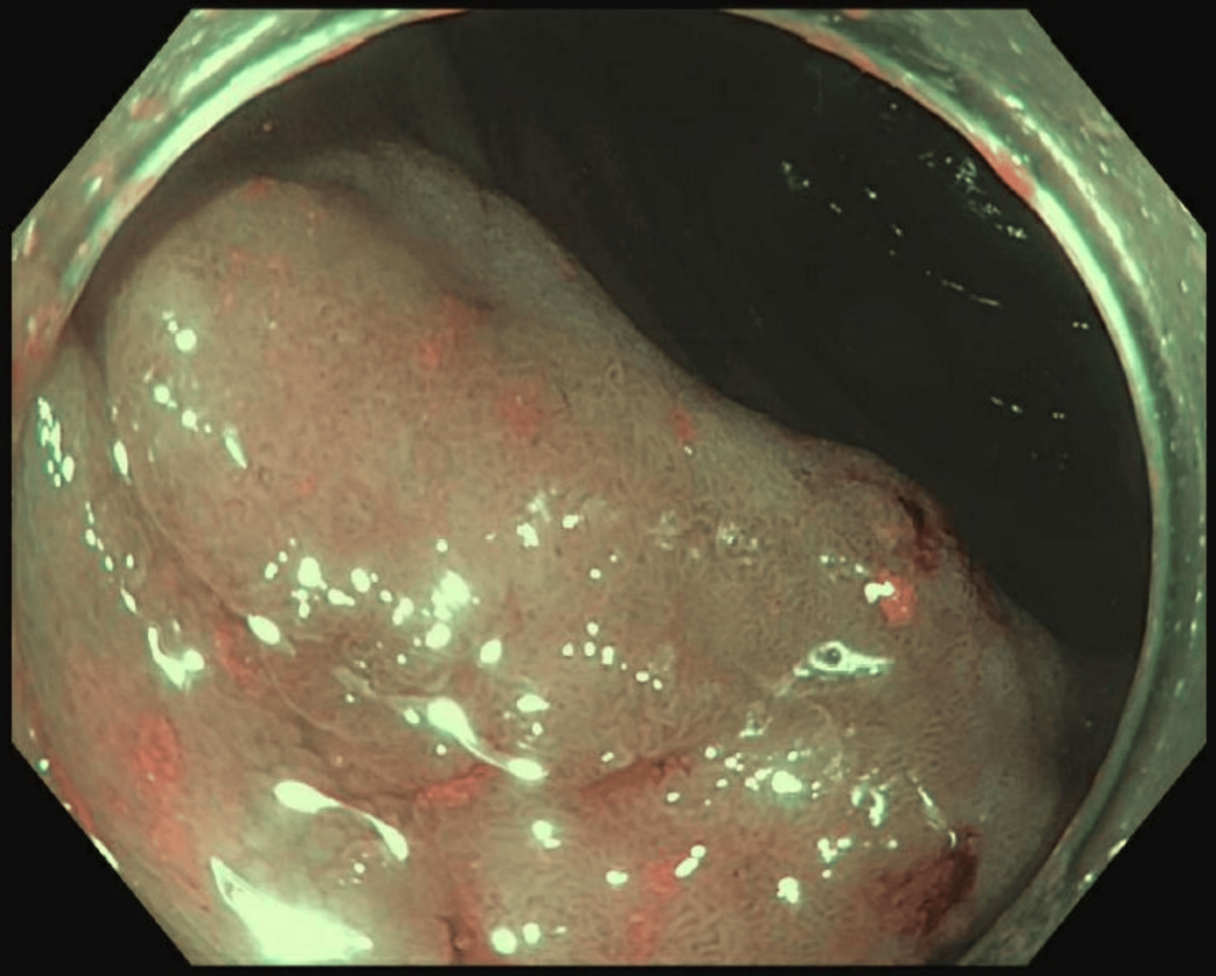
On endoscopy, a 30 mm sessile polyp was found in the ascending colon.

A 57-year-old male presented for routine surveillance colonoscopy and was found to have a 30 mm sessile polyp in the ascending colon (Figure 1).

**Figure 2:**
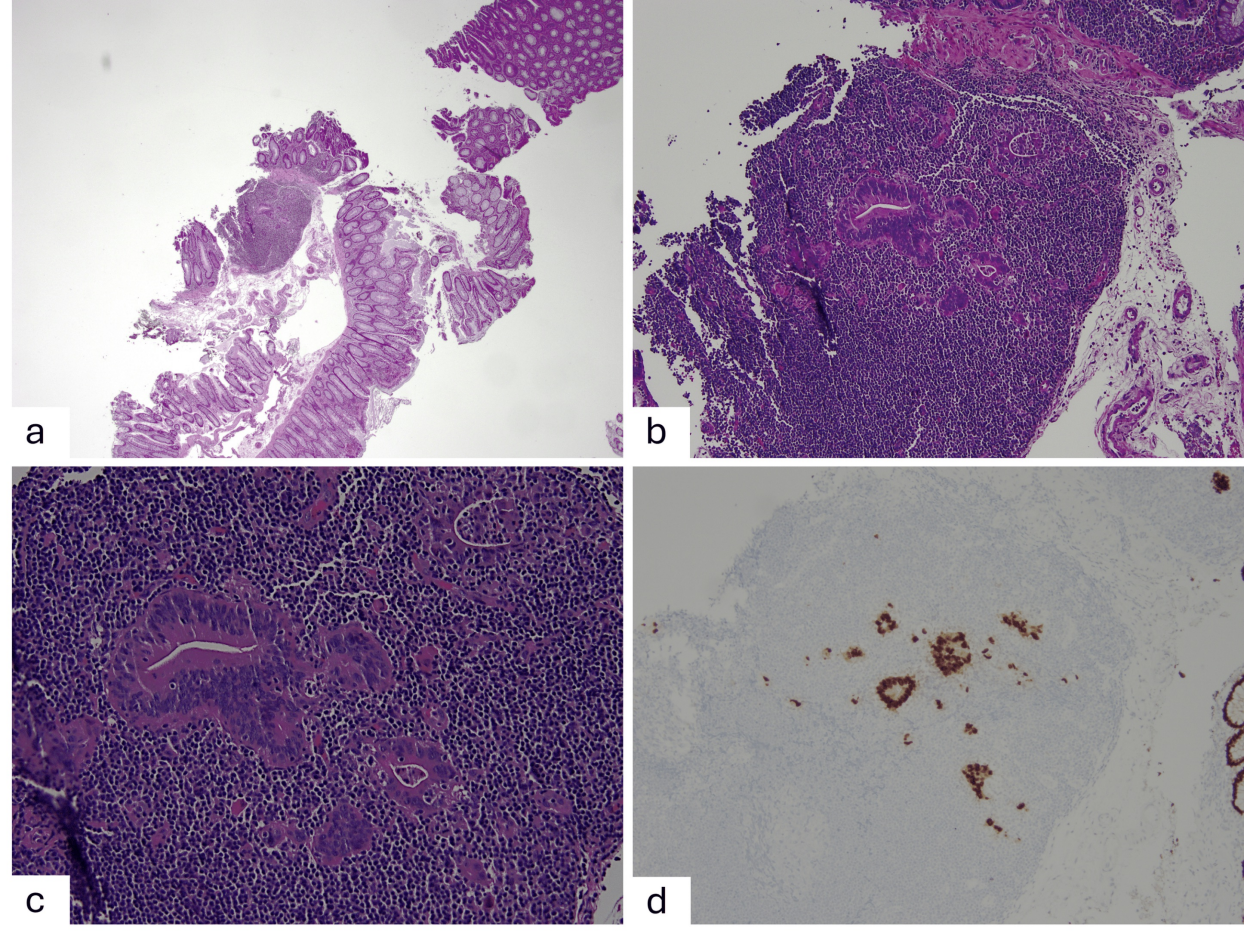
At low power, there is a small focus of tumor glands seen within a lymphoid aggregate in addition to the surface tubular adenoma (A, 20x). At higher power, the glands show haphazard distribution, gland angulation with luminal debris, scattered single cells, and high-grade cytologic atypia (B, 100x; C, 200x). CDX2 immunostain highlights the tumor glands and single cells within the lymphoid aggregate (D, 100x).

An endoscopic mucosal resection (EMR) was performed, and endoscopically, the post-resection margins were free of residual polyp tissue. The specimen was received in multiple fragments measuring 2.0 x 1.2 x 0.4 cm in aggregate and ranging in greatest dimension from 0.1 up to 1.9 cm. The surgical margins of the larger fragments were inked, and the inked fragments were sectioned. The tissue was entirely submitted in four blocks. Initial histologic sections show fragments of tubular adenoma. Due to the large size of the polyp, additional levels were ordered. At the second level of the largest fragment, there is a focus of lymphoid aggregate beneath the muscularis mucosa seen (measuring ~ 1.1 mm). Interestingly, there is a small focus of tumor glands (< 1 mm) within this lymphoid aggregate (Figure 2a). These glands are entirely surrounded by the lymphoid tissue and show an infiltrative pattern. There is no interposed lamina propria. The glands exhibit haphazard distribution, gland angulation with luminal debris, and scattered single cells. The cells show high-grade cytologic atypia (Figures 2b, 2c). Desmoplastic reaction is not seen. There is no lymphovascular or perineural invasion seen. CDX2 immunostain was performed and highlighted the tumor glands and single cells (Figure 2d), supporting the diagnosis of LGCC. The resection margin was not able to be confidently evaluated as the tissue was received in fragments. However, in the fragment that contains carcinoma, the inked deep resection margin was negative but close (< 1 mm), and the lateral margins were free of carcinoma in the fragment (Figure 2a). More levels were performed; however, the focus of carcinoma was only seen on three levels and disappeared on deeper levels. Additionally, focal high-grade dysplasia was seen in a different tissue fragment on a separate block.

This case was discussed at the multidisciplinary tumor board. Considering all the factors and the patient’s comorbidities (including BMI > 50, history of abdominal surgery), it was determined that the risk of surgery outweighs the benefits, and active surveillance was recommended. At six months follow-up, CT scans of the chest, abdomen, and pelvis showed no evidence of recurrent or metastatic disease. The serum CEA level was not elevated. At 10 months follow-up, repeat colonoscopy showed a post-mucosectomy scar at the EMR site with adjacent mucosal nodularity, which was biopsied and negative for dysplasia or malignancy.

## Discussion

Lymphoid aggregates are common throughout the gastrointestinal tract and frequently interact with the overlying mucosal epithelium. LGCC with malignant glands entirely confined by a lymphoid aggregate is uncommon and can resemble adenomatous polyps involving LGCs and pose a diagnostic challenge. Lee et al. compared the histologic findings of seven colonic adenomatous polyps involving submucosal LGCs to those of seven colorectal adenocarcinomas invading into submucosa (pT1) with associated submucosal lymphoid aggregates. They found that the main distinctive features of adenoma involving LGCs are glands with well-rounded contours contained within the lymphoid tissue; the consistent presence of lamina propria in the LGCs; and lack of infiltrating single cells/small clusters, poorly formed, fused, and irregular glands, solid tumor nest formation, desmoplastic reaction, and lymphovascular invasion [1]. In contrast, LGCC typically demonstrates irregularly distributed neoplastic glands with pronounced angulation or fusion; variable desmoplastic response; and occasional single cell infiltration. In LGCC, the malignant glands also lack interposed lamina propria and instead appear directly surrounded by lymphoid tissue. Desmoplastic stromal response can also be seen (Table 1) [2,3]. LGCC frequently arises in association with precursor polyps, including conventional adenomas and sessile serrated lesions, although rare de novo cases have been reported [2].

**Table 1:** Key histological features of LGCC versus its mimics. Source: [1,2]

Histological features	Lymphoglandular complex-like colorectal carcinoma (LGCC)	Adenomatous polyps involving lymphoglandular complexes (LGCs)	Pseudoinvasion/misplacement of adenomatous epithelium
Haphazard glands, angulation, fusion, or solid nest formation	Present	Not present	Not present
Lamina propria	Not present	Present	Present
Infiltrating single cells/small clusters	Can present	Not present	Not present
Desmoplastic stromal reaction	Can present	Not present	Not present
Lymphovascular invasion	Rare	Not present	Not present
Features of prolapse or prior biopsy	Not present	Not present	Commonly present

It is also important to differentiate LGCC from pseudoinvasion/misplacement of adenomatous epithelium secondary to prolapse or changes associated with prior biopsy. Pseudoinvasion/epithelial misplacement is most often associated with large pedunculated polyps and usually shows a lobular configuration with preserved lamina propria around the misplaced glands. Secondary changes, such as ruptured crypts, hemorrhage, hemosiderin deposition, and granulation tissue can be seen [1,4]. The misplaced glands usually maintain continuity with the surface adenoma and lack infiltrative features (Table 1) [1,5]. In some cases, immunohistochemical stains may also be helpful. It has been reported that relative to the overlying adenomatous component of the polyps, adenomas with adenocarcinoma often show significantly increased staining of the submucosal invasive epithelium and stroma for MMP-1, increased nuclear staining for p53, and decreased staining of the cytoplasmic membrane for E-cadherin. Collagen IV stain often shows discontinuous or complete absence of staining of the basement membrane in comparison with adenomas with misplaced epithelium [6].

The extent of carcinoma in LGCC is often limited, which can make it particularly challenging to diagnose. The malignant foci may only present on a few histologic levels for a large polyp. In our case, the initial section revealed only fragments of tubular adenoma. However, subsequent deeper sectioning showed a small lymphoid aggregate with a small focus of carcinoma (< 1 mm). The carcinoma was only present on three levels and disappeared on deeper levels. This underscores the importance of adequate histologic leveling for large colorectal polyps. The malignant focus was still present on the CDX2-stained level, and the stain highlighted single cells, which was helpful for the diagnosis in our case.

The pathogenesis of LGCC remains incompletely characterized due to its rarity. In a recent series of 20 cases from multiple centers, including mostly consult cases, the authors found that 90% of the cases are associated with a surface polyp, most commonly tubular adenomas (15 of 18 cases). The remaining three cases are associated with sessile serrated lesions with cytologic dysplasia [2]. Rare cases without identifiable precursor lesions have been reported, and it has been suggested that the carcinoma in these cases may emanate from an adenoma arising within LGCs [2].

The identification of invasive carcinoma in polyps, even when limited in extent, carries significant implications for patient management. For malignant polyps with high-risk features, such as positive margin, submucosal invasion > 1 mm, poorly differentiated morphology, lymphovascular invasion, tumor budding, and piecemeal resection, surgery may be indicated. However, location, comorbidities, and patient preference should also be considered when making the final decision, and a multidisciplinary approach has been advocated for an optimal outcome [7,8]. In our case, the polyp was removed through piecemeal resection. The focus of LGCC was only present in the largest tissue fragment, and the inked deep margin in this fragment was negative for carcinoma, but was close (< 1 mm). This case was thoroughly discussed at the multidisciplinary tumor board. Considering all the factors and the patient’s comorbidities (including BMI > 50, history of abdominal surgery), it was determined that the risk of surgery outweighs the benefits, and active surveillance was recommended. At 10 months follow-up, clinical, imaging, and endoscopy examinations show no evidence of recurrent disease or metastasis.

## Conclusions

LGCC is a very rare variant of colorectal adenocarcinoma characterized by malignant glands entirely enveloped by a prominent lymphoid aggregate. The carcinoma focus can be extremely small and difficult to identify. Deeper levels in large adenomas with prominent lymphoid aggregates can be helpful. LGCC can closely mimic adenomatous polyps involving LGCs or pseudoinvasion/misplacement of adenomatous epithelium. Awareness of the morphologic features can help to avoid diagnostic pitfalls.
